# Risk factors associated with scabies infestation among primary schoolchildren in a low socio-economic area in southeast of Iran

**DOI:** 10.1186/s12887-021-02721-0

**Published:** 2021-05-25

**Authors:** Alireza Sanei-Dehkordi, Moussa Soleimani-Ahmadi, Mehdi Zare, Seyed Aghil Jaberhashemi

**Affiliations:** 1 Social Determinants in Health Promotion Research Center, Hormozgan University of Medical Sciences, Bandar Abbas, Bandar Abbas, Iran; 2 Department of Medical Entomology and Vector Control, Faculty of Health, Hormozgan University of Medical Sciences, P.O. Box: 79145–3838, Bandar Abbas, Iran; 3 Department of Occupational Health Engineering, Faculty of Health, Hormozgan University of Medical Sciences, Bandar Abbas, Iran; 4 Bashagard Health Center, Hormozgan University of Medical Sciences, Bashagard, Iran

**Keywords:** Scabies, Prevalence, Risk factors, Primary schoolchildren, Bashagard, Iran

## Abstract

**Background:**

Scabies is a neglected tropical disease caused by the mite *Sarcoptes scabiei* that burrows under the skin. It is a major health problem in tropical areas, largely affecting children. Scabies is common and highly contagious and in schoolchildren spreads quite rapidly, due to overcrowding and close contact within the schools. This study aimed to determine the risk factors associated with scabies infestation among primary schoolchildren in Bashagard County, one of the low socio-economic areas in southeast of Iran.

**Methods:**

To conduct this community-based, descriptive, and cross-sectional study, four primary schools were randomly selected in the Bashagard County. All students in these schools were selected and examined for scabies. Clinical examination and sociodemographic profile of students were assessed using a pre-tested structured questionnaire. Chi-square test, and binary logistic regression were used to analyse the factors associated with scabies infestation by SPSS version 21 software.

**Results:**

Out of 480 studied schoolchildren, 15 cases of scabies with a prevalence of 3.1 % were observed. The frequency of infestation in males was 1.6 % and it was 4.7 % in females. Independent factors associated with a high risk of scabies infestation in unadjusted analysis were being student of grade 5–6 (cOR = 13.12, 95 % CI 2.92–58.89, *p* = 0.0001), low educational level of father (cOR = 4.37, 95 % CI 0.97–19.59, *p* = 0.036), low educational level of mother (cOR = 4.14, 95 % CI 1.92–18.57, *p* = 0.045), joblessness of father (cOR = 14.77, 95 % CI 4.97–43.89, *p* = 0.0001), employment of mother (cOR = 5.28, 95 % CI 1.38–20.16, *p* = 0.007), large family size (cOR = 3.34, 95 % CI 1.05–10.64, *p* = 0.031), use of shared articles (cOR = 33.37, 95 % CI 10.82–102.90, *p* = 0.0001), and absence of bathroom in the house (cOR = 11.77, 95 % CI 2.16–63.94, *p* = 0.0001).

**Conclusions:**

Results of this study confirmed that scabies is still one of the most important health problems in the primary schools of the Bashagard County. Low socioeconomic status and personal hygiene of the schoolchildren were the most important factors influencing the prevalence of scabies. Improvement of socioeconomic conditions and implementation of appropriate educational programs and active surveillance system to quickly detect and treat scabies cases are necessary in order to reduce the prevalence of scabies in schoolchildren in this area.

## Background

Scabies is a skin infestation caused by *Sarcoptes scabiei var. hominis*, an obligate parasite mite which burrows in the lower stratum corneum of the skin [1].

The annual world burden of scabies is estimated to be 300 million cases [2]. It is more prevalent in tropical and humid regions and was listed as a neglected tropical disease (NTD) by the WHO in 2013 [3]. The major clinical manifestations of scabies is a generalized pruritic rash, worsening at night [4]. Scabies is transmitted through close personal contact, as well as through infected clothing. Although it is associated with low morbidity, scabies can lead to dermatitis, intense itching, and secondary bacterial infections [5]. It can also cause psychological frustration and intense anxiety among the infected people [6]. The most common sites of infestation are the wrists, buttocks, fingers, genitals, axillae, groins, and the breasts in women. In young children and infants, the soles, palms, neck, and face are involved more commonly [7].

Epidemiological studies have shown that the prevalence of scabies infestations is not influenced by race, age, or sex and the main contributing factors are poor hygiene, poverty, and overcrowded living conditions [8].

Scabies affects all social classes, however, some groups of people, such as immunocompromised individuals, the elderly, residents of care facilities, children, and populations with low socioeconomic conditions, are at higher risk of infection [9].

Scabies is a common problem in schoolchildren and the infestation spreads rapidly due to close physical contact between classmates and overcrowding conditions in the schools [10, 11].

Scabies has a different epidemiological distribution among different communities [12]. In Iran, most of the epidemiological studies about scabies have been conducted among prisoners and military personnel. Results of these studies indicated that sociodemographic factors such as educational level, family size, and level of personal hygiene influence the prevalence of scabies in these populations. However, few studies have reported the prevalence of scabies and its related factors in schoolchildren [13]. Therefore, it is important to recognize the factors that may influence the prevalence of scabies to provide basic information for interventions toward the prevention and control of infestation in schoolchildren. This study aimed to determine the risk factors associated with scabies infestation among primary schoolchildren in Bashagard County, one of the low socio-economic areas in southeast of Iran.

## Methods

### Study areas

**Fig. 1:**
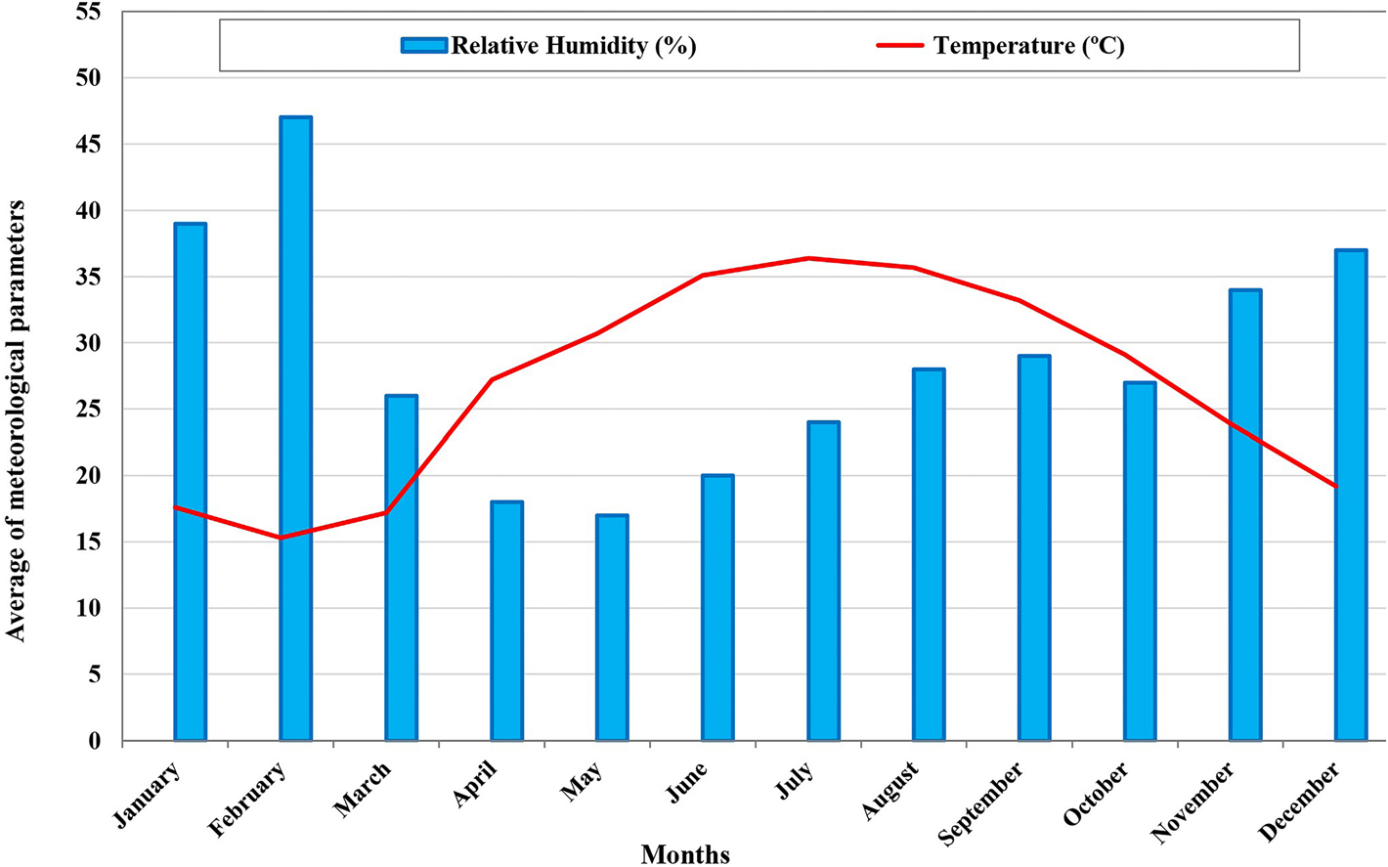
Average of meteorological parameters in Bashagard County, southeast Iran, 2018

This study was conducted in Bashagard County in the Hormozgan Province, southeast of Iran. This County has an area of 16,000 km^2^ and is placed between longitudes 57°23’-59°02’ E and latitudes 26°04’-26°58’ N, with 35,085 populations according to 2016 census, from which 51 and 49 % were males and females, respectively [14]. The area is hilly with low precipitation. It has a hot and dry climate. In this county, the average annual rainfall is 262 mm and the averages of maximum and minimum relative humidity are respectively 47 % in February and 17 % in May. The climate in this region is tropical with a mean annual temperature of 26.7 °C, ranging from 15.3 to 36.4 °C (Fig. 1).

According to the latest divisions of the country in 2016, Bashagard county has 2 city and 227 villages. The villages are scattered and small with low number of population and are located close to permanent and seasonal rivers [14]. This County is an undeveloped and remote area in the Hormozgan province. Most of the people in this area are poor and live in houses made of cement blocks and shelters.

### Study design and sample size

**Fig. 2:**
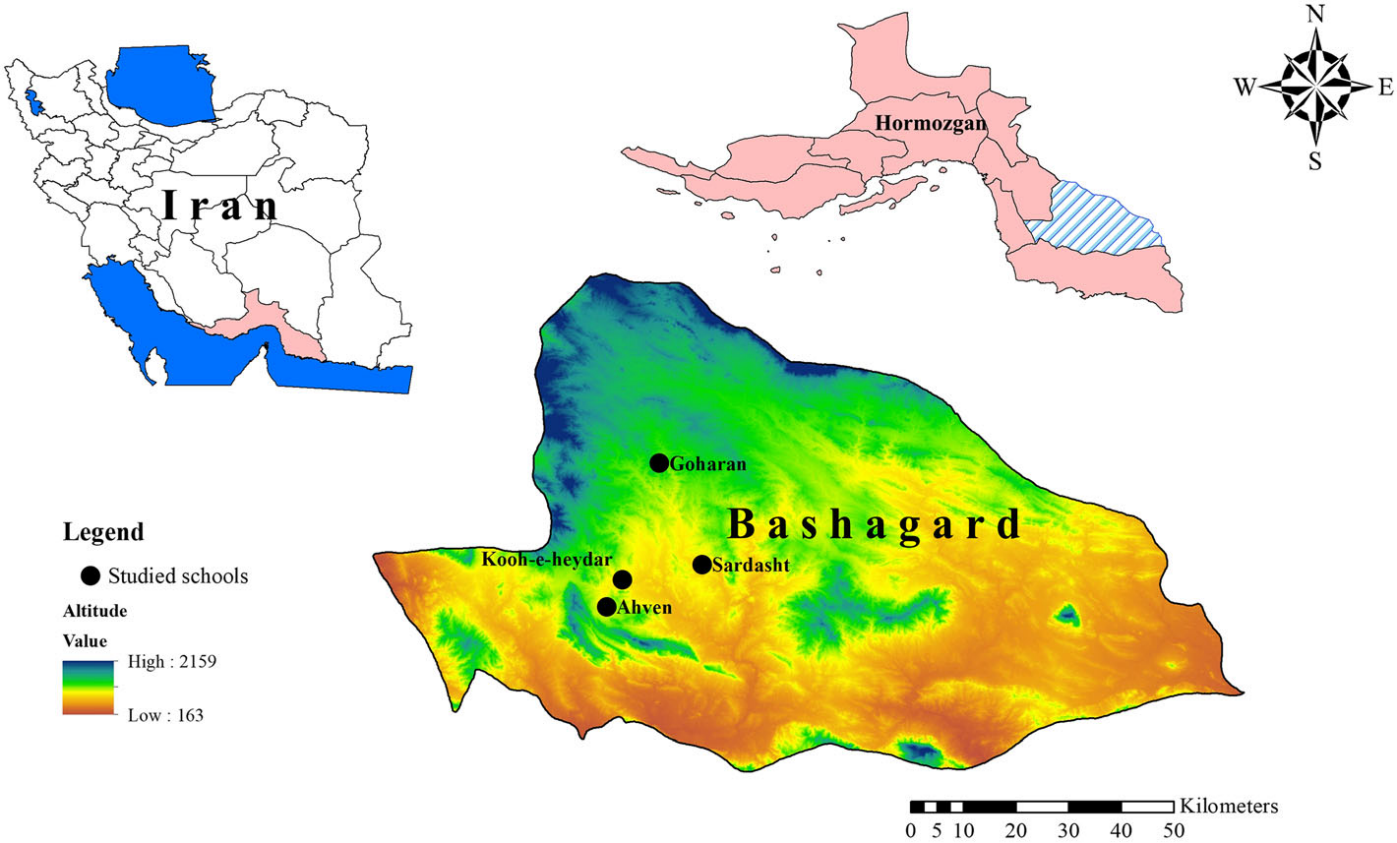
Map showing the location of study schools in Bashagard County, Hormozgan province, southeast Iran

On the basis of unpublished epidemiological data on the prevalence of scabies infestation in Bashagard health center, Bashagard County was considered to be investigated. In this regard, a community-based descriptive cross-sectional study was conducted from October to December 2018 in this County. To calculate the sample size, the maximum variability was assumed to be 7 % [15]. With 95 % confidence level and ± 2.3 % precision, the minimum number of subjects was determined using the formula (n = z^2^pq/d^2^) to be 472. A multi-stage cluster sampling method was used to select the primary schoolchildren. There were two cities and four villages in the Countiy that had the inclusion criteria and in the first stage all of them were considered as the clusters. In the next stage, two cities and two villages were selected according to their population. All of the schools and students in the selected clusters were considered as the study subjects. In this regard, two urban schools in Gouharan and Sardasht cities and two rural schools in Ahven and Kooh-e-heydar villages were enrolled in the study (Fig. 2).

All of the schoolchildren in the clusters were examined for scabies. Clinical examinations were performed in a private room for all of the students in each school by a trained physician. In the first step, suspected scabies cases were identified according to the presence of burrows or erythematous papular, vesicular, and pustular lesions with itching. In the next step, sampling of borrows of the patient by scraping test methods was used only in suspected cases to confirm the scabies infestation [10]. After the examination, all of the schoolchildren were interviewed by a structured questionnaire. Before using the questionnaire, its reliability and validity was tested. In this regard, content validity ratio (CVR) and content validity index (CVI) were used to validate the questionnaire [16, 17].The results of CVI and CVR were satisfactory for all items. In this regard, the CVI ranged from 0.82 to 1.00 and CVR ranged from 0.72 to 1.00). Internal consistency of the questionnaire was evaluated by calculating Cronbach’s alpha coefficient [18]. Test-retest reliability was also determined by completing the questionnaire twice during a two-week interval by 40 participants. The intra-class correlation coefficient (ICC) was calculated to determine the stability of the questionnaire over the time [19]. Internal consistency of the questionnaire and its test-retest reliability were also satisfactory (Cronbach’s alpha = 0.88 and ICC = 0.87).

The questionnaires were directed by trained interviewers and supervised by the principal investigator. The questionnaire included different items such as demographic characteristics, presence of health teacher in the schools, parent’s educational level, parent’s job, family size, bathroom availability in the home, and construction materials and water supply of living houses.

### Statistical analysis

To analyze the data, SPSS ver.21 software was used. Descriptive statistics were used to show averages, percentages, and relative frequencies. Chi-square test and binary logistic regression model were used to analyze the data. For every potential predictors of scabies infestation, odd ratio (OR) obtained from bivariate logistic regression model and 95 % confidence intervals (CI) were reported. The results were considered significant at 5 % level (*p*-value < 0.05).

### Inclusion and exclusion criteria

The inclusion criteria were being the students of a primary school with the students of different grades in separated classrooms and willing to participate in the study.

The exclusion criteria were unwilling to participate in the study and being absent at the time of visiting.

## Results

A total of 480 students including 257(53.3 %) males and 223(46.5 %) females were examined. The average of students’ age was 9.6 ± 1.77 years, ranging from 6 to 13 years. The mean family size was 5.4 ± 2.2 persons, ranging from 2 to 13 persons. Nearly 88 % of the schoolchildren were living in houses constructed using cement blocks. Majority of the schoolchildren had access to sanitary tap water (94.2 %), and all of them had electricity in their houses. In addition, 98.3 % of them had bathroom in their houses (Table 1).

**Table 1:** House characteristics of schoolchildren in Bashagard County, southeast Iran

Characteristics	Number	Precent
**Type of house**		
Cement- blockhouse	422	87.9
Muddy	44	9.2
Shed	14	2.9
**Water supply**		
Yes	452	94.2
No	28	5.8
**Electricity**		
Yes	480	100
No	0	0
**Bathroom availability in house**		
Yes	472	98.3
No	8	1.7

Majority of the fathers of schoolchildren (60.6 %) were either illiterate or had a primary level of education, also, 93.1 % of the children’s fathers were employed. In addition, most of the children’s mothers (61.9 %) were either illiterate or had only received a primary education, and 95 % were housewives (Table 2).

**Table 2:** Sociodemographic characteristics associated with scabies infestation in schoolchildren of Bashagard County, southeast Iran

Characteristics	Total examined cases		Positive cases		Crude OR (95 % CI)	*p*-value
	No.	%	No.	%		
Sex						
Males	257	53.5	4	1.5	1	0.034
Females	223	45.5	11	4.9	3.28 (1.03–10.45)	
School grade						
1–4	313	65.2	2	0.6	1	0.0001
5–6	167	34.8	13	7.78	13.12 (2.92–58.89)	
Father’s education						
Illiterate/ Primary	291	60.6	13	4. 4	1	0.036
Secondary/ High school/ University	189	39.4	2	1	4.37 (0.97–19.59)	
Mother’s education						
Illiterate/ Primary	297	61.9	13	4.4	4.14 (1.92–18.57)	0.045
Secondary/ High school/ University	183	38.1	2	1.1	1	
Father’s Job						
Employee/ Farmer/ Self-employment	447	93.1	8	1.8	1	0.0001
Jobless	33	6.9	7	21.2	14.77 (4.97–43.89)	
Mother’s Job						
Employee	24	5	3	12.5	5.28(1.38–20.16)	0.007
Housewife	456	95	12	2.6	1	
Family size						
2–4	259	53.9	4	1.5	1	0.031
> 4	221	46.1	11	4.8	3.34(1.05–10.64)	

*OR* Odds ratio, *CI* Confidence interval

**Fig. 3:**
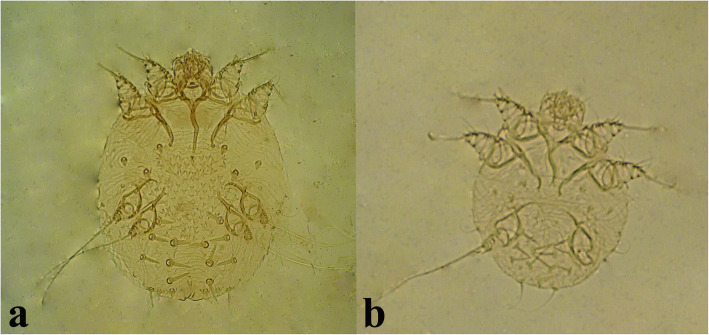
Scabies mite detected on the scrapings of the skin legions in schoolchildren of Bashagard County, south of Iran. Adult mite (**a**), Larvae (**b**)

In this study *Sarcoptes scabiei* (Fig. 3) was detected on the skin lesions of 15 out of 480 schoolchildren. Accordingly, the prevalence of scabies among schoolchildren was 3.1 %. The frequency of infestation in male and female students were 1.5 and 4.9 %, respectively, and females were more likely than males to be infected by scabies (OR = 3.28, 95 % CI 1.03–10.45, *p* = 0.034; Table 2).

**Fig. 4:**
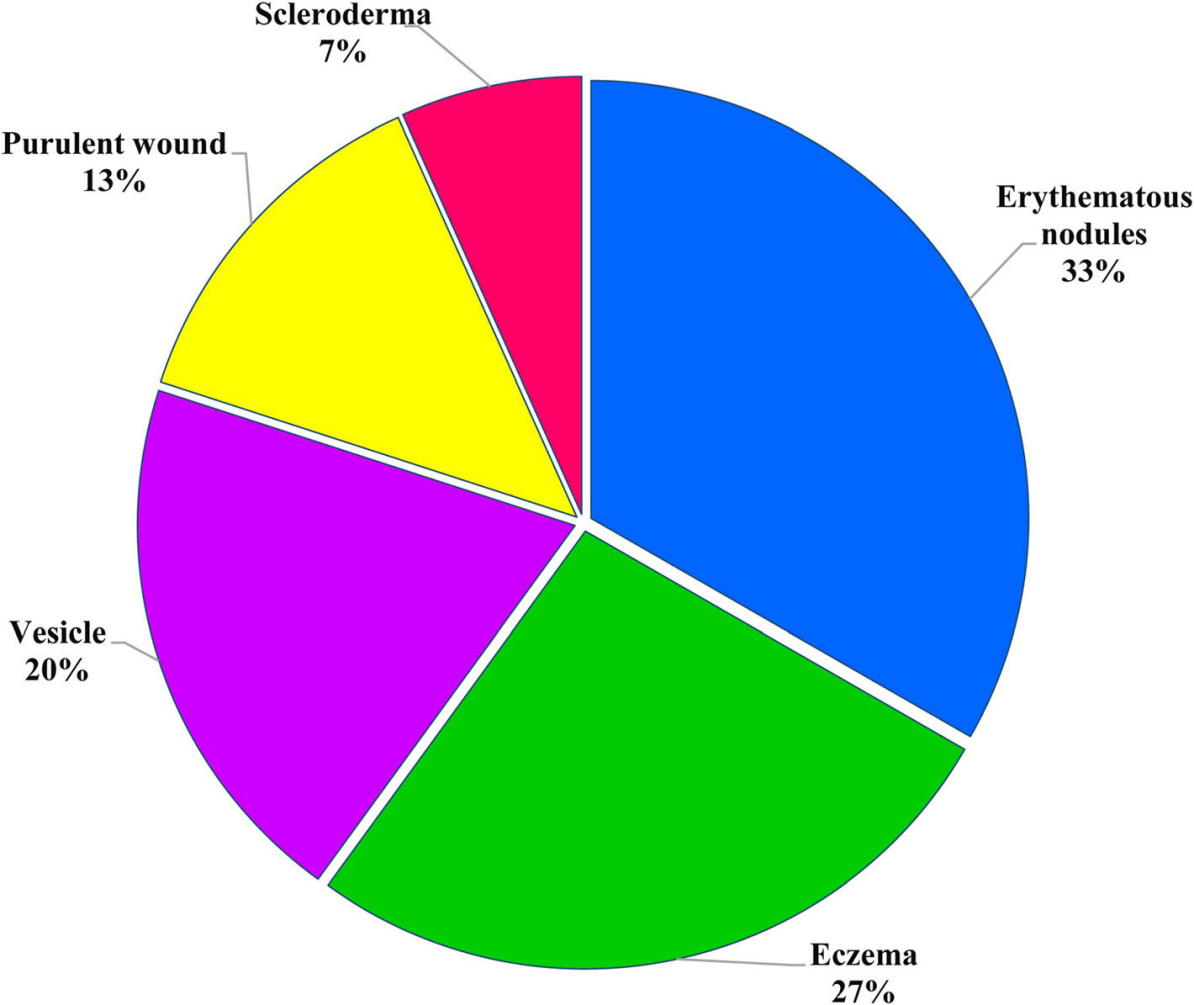
Various forms of scabies lesions in schoolchildren of Bashagard County, southeast Iran

Itching was the most prevalent symptom (84.6 %), with 64 % presenting severe itching and 49 % complained of itching-related sleep disturbance. Moreover, Erythematous nodules were the most common skin lesions in the students (Fig. 4).

**Fig. 5:**
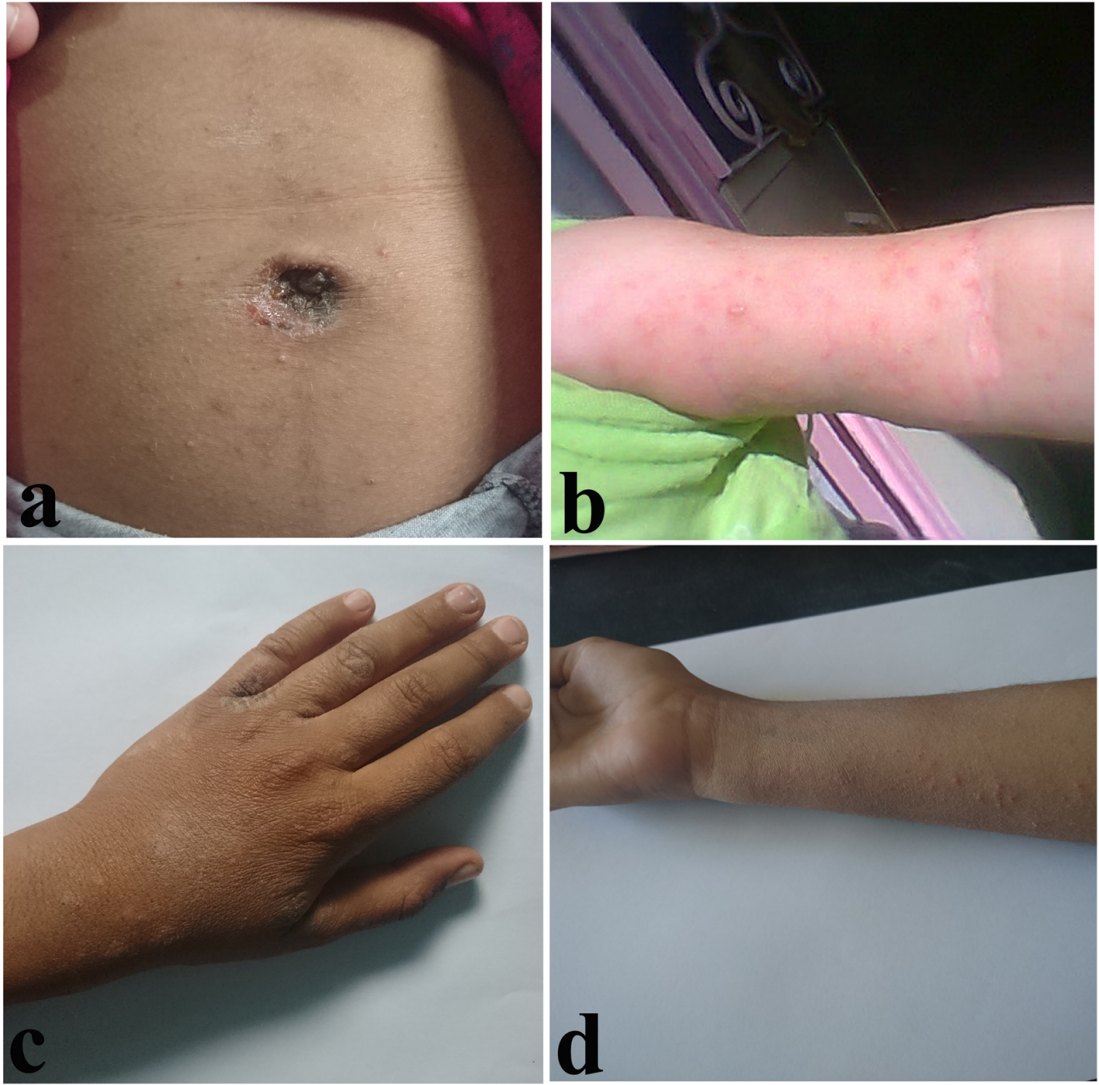
Locations of scabies infestation in schoolchildren of Bashagard County, southeast Iran. abdominal area (**a**), arm (**b**), web spaces between the fingers (**c**), and forearm (**d**)

As Fig. 5 indicates, lesions were observed on the web space between the fingers (33.3 %), wrists (20 %), abdomen (13.3 %), forearms and arms (13.3 %), thighs (6.7 %), anterior axillary folds (6.7 %), and legs (6.7 %).

The study showed that the prevalence of scabies was significantly higher in the grade 5–6 students and the students in these grades were more likely to develop scabies than grade 1–4 students (OR = 13.12, 95 % CI 2.92–58.89, *p* = 0.0001; Table 2).

The study results also indicated that the prevalence of scabies was significantly related to the parents’ educational level. In this regard, lower levels of education for either the father or the mother were found to increase the risk of scabies significantly (OR = 4.37, 95 % CI 0.97–19.59, *p* = 0.036 for paternal education; and OR = 4.14, 95 % CI 1.92–18.57, *p* = 0.045 for maternal education; Table 2).

According to the results, scabies infestation was significantly related to the parents’ job. The risk of scabies infestation was higher in schoolchildren whose fathers were jobless (OR = 14.77, 95 % CI 4.97–43.89, *p* = 0.0001), and the students with employed mothers had higher risk of infestation (OR = 5.28, 95 % CI 1.38–20.16, *p* = 0.007; Table 2).

In this study, infestation rate of scabies was positively associated with family size and the risk of scabies infestation was higher in the crowded families with more than four members (OR = 3.34, 95 % CI 1.05–10.64, *p* = 0.031; Table 2).

The study results also showed that the prevalence of scabies infestation was 31 % among schoolchildren who used shared articles such as towel, combs, and cloth and they were more likely to be infested by scabies than students who did not use shared articles (OR = 33.37, 95 % CI 10.82–102.90, *p* = 0.0001; Table 3). In addition, the prevalence of scabies infestation was higher among students with shared bedroom compared to those with private bedroom. However, sharing the bedroom was not found to affect the risk of infestation significantly (OR = 1.76, 95 % CI 0.23–13.68, *p* = 0.58; Table 3).

**Table 3:** Behavioral characteristics associated with scabies infestation in schoolchildren of Bashagard County, southeast Iran

Characteristics	Total examined cases		Positive cases		Crude OR (95 % CI)	*p*-value
	No.	%	No.	%		
Use of shared articles						
Yes	29	6.1	9	31.0	33.37(10.82–102.90)	0.0001
No	451	93.9	6	1.3	1	
**Sharing bedroom**						
Yes	427	89.2	14	3.3	1.76(0.23–13.68)	0.583
No	53	10.8	1	1.9	1	
**Bathroom availability in house**						
Yes	472	98.3	13	2.7	1	0.0001
No	8	1.7	2	25	11.77(2.16–63.94)	

*OR* Odds ratio, *CI* Confidence interval

In the present study, the risk of scabies infestation was significantly higher among the students who did not have bathroom in their houses compared to those who had bathroom (OR = 11.77, 95 % CI 2.16–63.94, *p* = 0.0001; Table 3).

## Discussion

Epidemiological studies about scabies infestation provide valuable information about the associated risk factors and serve as a basis for selection of prevention methods and control and therapeutic services. According to the results of this study, the prevalence of scabies was 3.1 % in primary schoolchildren of Bashagard County, with higher infestation rate in female students (4.7 %), compared to that of males (1.6 %). Previous studies have reported the prevalence of scabies infestation in primary schoolchildren from 2.09 to 7.22 % in different parts of Iran [13, 20–23]. In similar studies, conducted on schoolchildren of Egypt, Nigeria, Turkey, and, Kuwait, the prevalence of scabies was 4.4 %, 4.8 %, 2.16 %, and 3 %, respectively [24–27]. In addition, studies from India and Cameron have reported high infestation rate of scabies among schoolchildren with the prevalence of 39.42 and 17.8 %, respectively [28, 29]. The variation of scabies infestation rate may be due to different factors such as family size, personal hygiene, and economic conditions [30, 31]. Prevalence of scabies infestation in the study area can be attributed to factors such as use of shared articles, low parents’ educational level, large family size, low frequency of bathing per week, and poor health facilities. Obviously, many of these factors are the result of extreme poverty.

In this study, the most common locations of scabies lesions were the web spaces between the fingers and wrists. A similar study in the north of Iran also reported the highest number of scabies infestation on the web spaces between the fingers [22]. Scabies infections are usually localized in specific parts of the body and elbows, wrists, and hands are the most commonly infected sites [32]. Hand and wrist infection may occur due to handling mite-contaminated materials and touching infected persons. However, the distribution patterns suggest that the mites select special locations of the body and these locations may be preferred partly according to the lipid composition [32, 33].

In this study the highest prevalence of scabies infestation was observed in the grade 5–6 students and in children aging more than 10 years. This finding is in accordance with the results of previous studies in Brazil, Nigeria, Pakistan, and Sri Lanka [7, 34–36]. This can be explained by the more direct physical contact with friends at this students’ age group. In this regards, other studies also have reported the important role of physical contacts in transmission of scabies [35].

According to the results, scabies was more prevalent among schoolchildren with low educated parents. Similar findings have been reported from Iran [22, 23, 26, 37–40]. Parental education seems to have a major role in prevention of contagious diseases. Many studies have reported that parents with higher levels of education are more capable to apply healthcare and prevention measures for their children [26, 39–41]. Since educational-based interventions have been reported to be efficient in reduction of insect-borne diseases in low socioeconomic areas [42], it is essential to provide appropriate educational programs for teachers, parents, and students to increase their awareness about scabies infestation risk factors and related prevention measures [4].

The results of the present study have confirmed the positive relationship between scabies infestation and the family size. This finding is supported by previous studies in Iran, Saudi Arabia, Cameroon, Ethiopia, Fiji, Mali, Malawi, and Cambodia [21–23, 29, 38, 43–45]. Larger family size leads to overcrowding. In the overcrowded homes, close contact between family members and use of shared beds and cloths, increases the risk of scabies transmission. In addition, having more children may result in higher scabies infestation rates, because parents with more children pay less time per child to perform laundry and other personal hygiene activities.

According to the results of this study, use of shared articles such as towel, combs, and cloth affects the scabies prevalence. In a similar study conducted by Karim et al. in Bangladesh [40], children who had shared beds on the floor, contracted more severe scabies infections and became re-infected more frequently. It was attributed to the use of shared beds which facilitates skin-to-skin contact and transmission of scabies from infested children to healthy ones. Similar findings have been reported from Egypt, Pakistan, Brazil, Ethiopia, Argentina, and Taiwan [26, 35, 39, 46–48]. In the crowded conditions, use of shared beds, cloths, and other materials may transmit the scabies infestation [48].

The present study revealed that the prevalence of scabies was higher among schoolchildren who had no bathroom in their homes. This finding is similar to the results of Wochebo et al. study in Ethiopia who reported the risk of scabies is significantly associated with personal hygiene and children who took bath less than two times a week were five times more likely to be infected by scabies [46]. Likewise, in previous studies conducted in Ethiopia, Nigeria, Saudi Arabia, Bangladesh, and Cameroon an association between scabies infestation and frequency of bathing was reported [11, 34, 38, 40, 42]. This might be because primary school students have less control over their personal hygiene. In addition, since schoolchildren spend much of their time with their friends, they have a higher chance of physical contact which facilitates scabies transmission. Other studies also have confirmed that the prevalence of human scabies is linked with poor personal hygiene [10, 49]. Therefore, the prevalence of scabies infestation in primary school children may be, to some extent, due to low awareness and practice regarding the personal hygiene.

## Conclusions

Results of this study confirmed that scabies is still one of the most important health problems in the primary schools of Bashagard County.

Low parents’ educational level, father’s joblessness, employment of mother, large family size, use of shared articles such as towel, comb, and cloth, absence of bathroom in the house, and poor health facilities were recognized as significant sociodemographic factors associated with prevalence of scabies among primary schoolchildren in the study area. Implementation of appropriate health education programs for students and their parents is necessary to increase their awareness about scabies risk factors and the preventive measures for successful management of scabies in schoolchildren. In addition, implementation of an active surveillance system for early case detection by medical staff and periodic clinical check-ups for schoolchildren to provide early treatment, particularly in rural areas, is necessary.

### Limitations of the study

This study was conducted only during the autumn season, and since scabies is claimed to show higher incidence during the winter months, the findings cannot be used to determine the annual prevalence of scabies among schoolchildren in the study area. Moreover, multi-variable analysis could not be conducted due to low prevalence of disease among the sub-categories. 

## Data Availability

The data are available from the corresponding author on reasonable request.

## Abbreviations

NTD: Neglected tropical disease
OR: Odds ratio
CI: Confidence interval
CVR: Content validity ratio
CVI: Content validity index
